# The relationship between the level of vitamin D and ruptured intracranial aneurysms among patients with high sun exposure

**DOI:** 10.1038/s41598-024-53676-y

**Published:** 2024-02-12

**Authors:** Lívio Pereira de Macêdo, Renata de Castro Tavares, Mateus Torres Braga, Lidiane Moura dos Santos, Glaudir Donato, Fábio Antônio Serra de Lima Júnior, Rosanne Pereira de Macêdo, Arlindo Ugulino Netto, Kauê Franke, Pierre Vansant Oliveira Eugênio, Auricélio Batista Cezar-Junior, Igor Vilela Faquini, José Laércio Júnior Silva, Eduardo Vieira de Carvalho Júnior, Nivaldo S. Almeida, Francisco Alfredo Bandeira e Farias, Marcelo Moraes Valença, Hildo Rocha Cirne Azevedo-Filho

**Affiliations:** 1 Department of Neurosurgery, Hospital da Restauração, Recife, Pernambuco Brazil; 2 Universidade Católica, Recife, Pernambuco Brazil; 3 Uninassau- Pernambuco, Recife, Pernambuco Brazil; 4 Medical Student, Centro de Ciências Médicas, Universidade Federal da Paraíba, João Pessoa, Paraíba Brazil; 5 Unifacid- Wyden, Teresina, Piauí Brazil; 6 Hospital Agamenon Magalhães, Recife, PE Brasil; 7 Universidade Federal de Pernambuco, Recife, Pernambuco Brazil; 8 Recife, Brasil

**Keywords:** Serum vitamin D, Aneurysmal subarachnoid hemorrhage, Tropical zone, Endocrinology, Neurology

## Abstract

Non-traumatic subarachnoid hemorrhage (SAH) accounts for 3–5% of acute strokes. Intracranial aneurysm is the most common cause of non-traumatic SAH. Vitamin D influences the cardiovascular system, including the formation and rupture of cerebral aneurysms. To evaluate the serum vitamin D level in patients living in the tropical zone who suffered aneurysmal subarachnoid hemorrhage and its correlation with demographic and neurological characteristics. This is an analytical cross-sectional study to assess the serum level of vitamin D in a study population of 99 patients treated and diagnosed with aSAH in a public hospital in Recife-PE over a period of 12 months. In the study sample, composed of individuals with high sun exposure due to the lifestyle they lead in a tropical region, we observed hypovitaminosis D (85.9%), with a median of 19.9 ng/ml, although the majority of individuals are skin with high concentration of melanin (Fitzpatrick skin type IV and V). In addition, rates of sun exposure are high to all patients (Solar Index 9.03 P50). Most individuals were female (79.8%); there was no statistical difference in solar exposure/solar index between genders. As for the neurological repercussions, there was no statistical relevance in the clinical prognostic scales evaluated. As the sample was composed mainly of individuals whose economic activity is agriculture, the values of solar index found are vastly higher than those of other studies conducted in high latitude regions. In line with the literature review, some aspects were raised with the objective of justifying such findings that go from the base of the poor diet of these individuals, the increase of melanin in the skin and genetic alterations that directs us to possible mechanisms of natural photoprotection to high sun exposure. Thus, we had a vast majority (85%) of hypovitaminosis D, which in fact makes us wonder if there is any influence of calcitriol on vitamin D receptors in vascular walls and in the cardiovascular system as a whole, which influence bleeding events of this nature. As for the neurological repercussions, measured using assessment scales (Glasgow coma scale, WFNS scale, Hunt–Hess and Fisher's tomographic scale) there was no significant difference in the results. As it is only a descriptive study, the causal relationship of the facts cannot be established. However, in a population exposed to high sun exposure and affected by aneurysmal SAH, there is a significant rate of hypovitaminosis D, which supports the hypothesis that vitamin D plays a role in vascular pathologies, such as cerebral aneurysms and SAH.

## Introduction

Intracranial aneurysms are present in approximately 1–2% of the population, with a rupture incidence of about 2–20 cases per 100,000 individuals per year. The initial hemorrhage causes death in up to 25% of patients before medical attention. Within 30 days, mortality reaches approximately 50%, and roughly half of the survivors exhibit severe neurological sequelae that impose significant physical and cognitive limitations. As such, aneurysmal subarachnoid hemorrhage (aSAH) is a significant medical emergency, associated with high morbidity and mortality^1^. The formation of intracranial aneurysms, as well as their rupture, depends on various factors, but the etiology remains incompletely explained^2–4^.

Vitamin D was first discovered in 1916 by Harry Steenbock of the University of Wisconsin in research aimed at curing rickets^5^. The endocrinological system of vitamin D encompasses various molecules within the vitamin D group, comprising secosteroid molecules derived from 7-dehydrocholesterol, both in the main pathway and in alternative (non-canonical) pathways. This includes the carrier protein (DBP) and its nuclear receptor (VDR). The primary pathway of vitamin D consists of a cascade of photolytic and enzymatic reactions starting from a cutaneous precursor derived from cholesterol (7-dehydrocholesterol) present in the deep layers of the epidermis (between the bilipidic layers of the stratum basale and spinosum). The active metabolite obtained from this process is considered a steroid hormone, 1,25-dihydroxyvitamin D. Conceptually, vitamin D is a prohormone, with its main endogenous source being cutaneous photobiosynthesis, remaining inert until it transforms into its active form, calcitriol (or 1,25-hydroxyvitamin D). The final reaction in the cascade for the formation of the active hormone occurs through the hydroxylation of 25-hydroxyvitamin D (calcidiol) by the enzyme 1α-hydroxylase, primarily taking place in the kidneys.

Recent studies have demonstrated non-canonical pathways of vitamin D activation that bypass the liver and are transported to organs expressing CYP11A1, including adrenals and other extra-adrenal tissues. In these alternative pathways, vitamin D3, lumisterol, tachysterol, and 7DHC serve as substrates for the activity of CYP11A1, which, either alone or in cooperation with other CYP enzymes, produces the corresponding hydroxyderivatives. The metabolites produced by non-canonical activation of vitamin D and activation of lumisterol and tachysterol are biologically active in ex vivo and in vivo experimental models and are non-toxic and non-calcemic at suprapharmacological doses. These metabolites can act on the vitamin D receptor (VDR) and on alternative receptors, including organ receptors. Thus, the fascinating role of CYP11A1 in the production of various hydroxyderivatives is emphasized. These derivatives, identified as biologically active compounds, underscore the complexity and versatility of vitamin D activation in the body. This implies the dynamic nature of vitamin D activation in various tissues throughout the body, providing an explanation for the observed pleiotropic effects of the D3 prohormone. They also challenge the current consensus conveyed by most literature that the main biologically relevant phenotypic effects of D3 can be exclusively attributed to the activation of VDR by 1,25(OH)2D3^6–10^.

The literature mainly focuses on the role of vitamin D in osteometabolism. However, recent studies emphasize the importance of vitamin D in other bodily systems. This is justified by the expression of vitamin D receptors (VDR) in all nucleated cells, including cardiomyocytes, vascular smooth muscle cells, and endothelial cells. Furthermore, vitamin D affects inflammation, cell proliferation, and differentiation^11–13^. Vitamin D receptors play an important role in the expression of vascular endothelial growth factor and metalloproteinase enzymes that affect vessel development and remodeling, in addition to having antiproliferative effects on smooth muscle cells in the arterial wall and a potent anti-inflammatory effect. This makes it crucial for maintaining the proper functioning and homeostasis of vascular endothelial cells^14–17^.

Vitamin D deficiency is also associated with increased expression of tumor necrosis factor-alpha (TNF-α) and hypoxia-inducible factor-1 alpha (HIF-1α), suggesting the protective role of vitamin D in regulating inflammatory mediators and hypoxia signaling against pathologies in which the inflammatory process plays a role. Several studies have attempted to correlate vitamin D deficiency with the increased prevalence of vascular diseases, including the formation and rupture of aneurysms^18–21^.

Studies on the role of vitamin D in the cardiovascular system have intensified since VDRs were found in cardiomyocytes^22–24^, vascular smooth muscle cells^25,26^, endothelial cells^27^, circulating monocytes, macrophages, dendritic cells, activated T lymphocytes^28^, and platelets.^29^ There is a strong association between low vitamin D levels and arterial disease, independent of traditional cardiovascular risk factors and regardless of the type of vascular disease, whether occlusive or aneurysmal^15,18,30–33^.

Regarding the correlation between vitamin D serum levels and intracranial aneurysms, it was only in the second half of the last decade that studies began to show a high incidence of vitamin D deficiency in patients undergoing cerebral aneurysm treatment, which persisted even after controlling for cardiovascular risk factors and smoking^34^. This study provided additional support for the concept that vitamin D deficiency is a factor to be considered in relation to the risk of aneurysm growth and rupture. Other studies have also demonstrated a high incidence of vitamin D deficiency in patients with cerebral aneurysms^34–37^. Among these, the study conducted by Randhawa and colleagues (2019) stands out as having the largest sample, confirming this relationship^35^. This correlation was questioned after observing the seasonality of serum vitamin D levels and aSAH throughout the year in some studies. These studies observed both a direct association of low vitamin D levels with hours of sunlight exposure, with a higher incidence of insufficiency in the winter months, and an independent relationship between aSAH and cold temperatures^38–40^.

Regarding the pathophysiology, factors present in the context of hypovitaminosis D, such as pro-oxidative and pro-inflammatory effects, decrease the immune response and increased blood pressure and endothelial and blood–brain barrier dysfunction, which may be the main reasons for the association of hypovitaminosis D with hemorrhagic stroke^41,42^. Although there are limitations in the studies, advances in research show a promising correlation. More research, both preclinical and clinical in nature, needs to be conducted.

**Figure 1:**
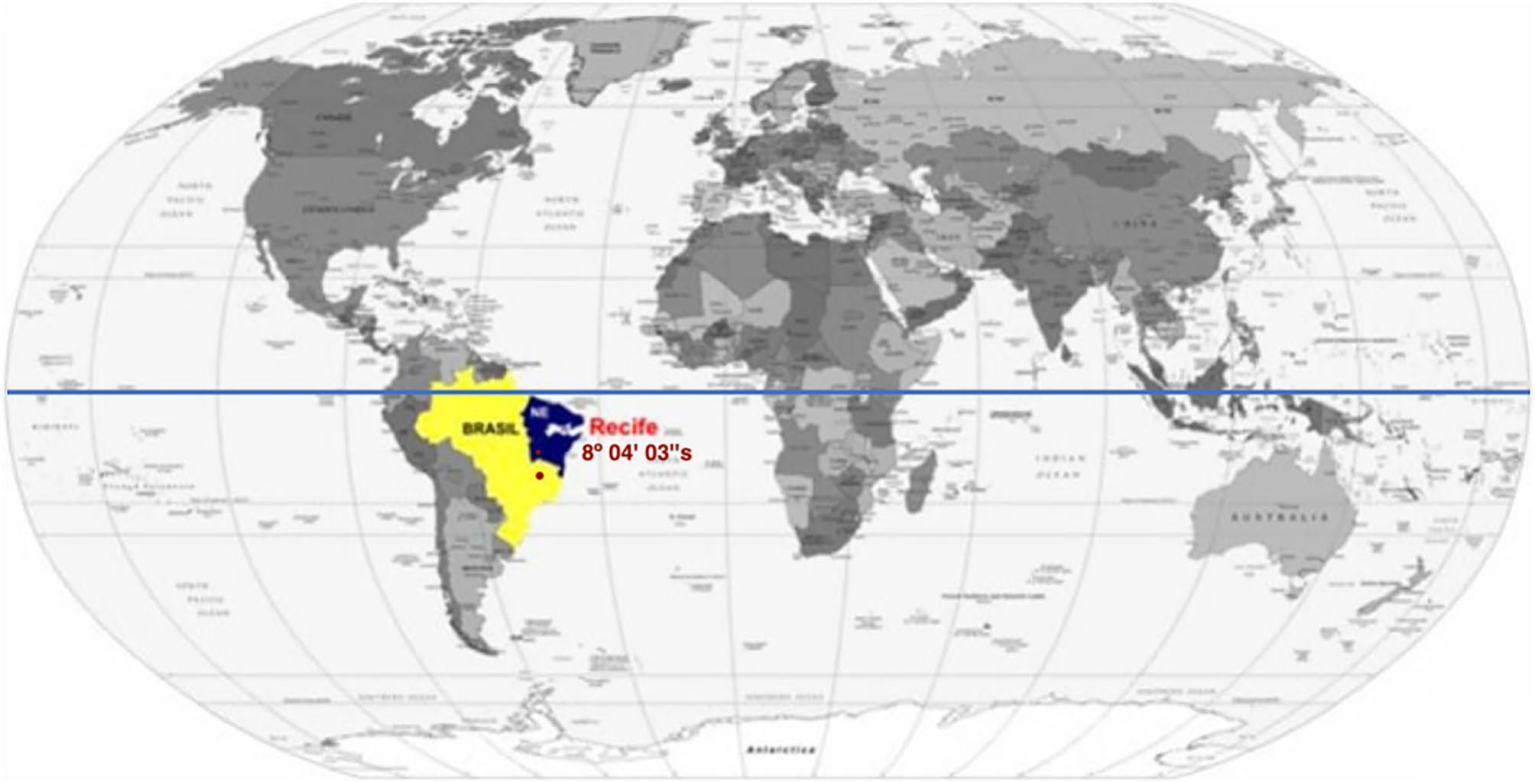
Low Latitude of Recife, Pernambuco, Demonstrated on the World Map.

In summary, regarding the relevance of the topics addressed in this research, it is important to note that between 1922 and August 2023, 41,102 articles mentioning the word "vitamin D" in the title were published on PubMed. It is noteworthy that 23,999 of these articles were published in the last 10 years, revealing the growing importance of this topic (PubMed, 2023)^43^. In a timeline overview, from its discovery in 1916, studies related to vitamin D sought to detail the role of vitamin D in bodily osteometabolism^5,44^. With the advancement of research and understanding of the extraskeletal action of this vitamin, research into the role of hypovitaminosis D in the pathophysiology of extraskeletal diseases, mainly cardiovascular and cerebrovascular diseases, intensified over the last 3 decades^16,17,45–50^. Only in the last decade, and especially in the past 7 years, research attempts have emerged to correlate vitamin D deficiency with cerebral aneurysms and subarachnoid hemorrhage (SAH)^34–37,51^. To date, all studies that correlate hypovitaminosis D with cerebral aneurysms and subarachnoid hemorrhage have been conducted in temperate climate countries. Therefore, here, we assess serum vitamin D levels in patients with ruptured cerebral aneurysms in a tropical region at a low latitude with high sunlight exposure (Fig. 1).

## Methodology

This is a cross-sectional study conducted with patients diagnosed with aneurysmal subarachnoid hemorrhage (aSAH) seen at the Neurosurgery Emergency Department of Hospital da Restauração in Recife, Brazil, from May 2021 to May 2022. In total, 99 patients were included in the study. The research was initiated and conducted after approval by the Hospital da Restauração’s Ethics Committee under CAEE (Certificate of Presentation of Ethical Assessment) number: 26467419.1.0000.5198. All methods were performed in accordance with the relevant guidelines and regulations. This research was performed in accordance with the Declaration of Helsinki. All our raw data and datasets used and analyzed during the current study are available from the corresponding author upon reasonable request.

### Inclusion criteria

Patients of both genders, aged 18 years or older, who had suffered from aneurysmal subarachnoid hemorrhage (aSAH), diagnosed through invasive or non-invasive neuroimaging examinations (magnetic resonance angiography or angiography) at a major hospital in Recife, Brazil, a reference in Neurosurgery, during the 12-month study period (May 2021 to May 2022) were included.

### Exclusion criteria

Exclusion criteria were as follows: (1) a known family or personal history of spontaneous aSAH or arteriovenous malformations based on previous imaging examinations; (2) chronic kidney or liver disease; (3) treatment with vitamin D for any reason; (4) pregnancy at the time of the analysis; (5) previous use of oral or injectable anticoagulants.

### Data collection

Clinical data were collected through patient interviews or with their family members and from medical records of visits to the Neurosurgery Emergency Department at Hospital da Restauração, Recife, Brazil, from May 2021 to May 2022. Informed consent was obtained from all subjects and/or their legal guardians in cases where the patient had an altered level of consciousness.

Peripheral blood samples were collected for laboratory serum 25-hydroxyvitamin D assays. The evaluation of skin phototypes followed Fitzpatrick's definition^52,53^, and the analysis of the Solar Index for each individual was based on the “Modified Rule of 9” described by Barger-Lux and Heaney^54^.

The anatomical severity of subarachnoid hemorrhages was characterized according to the Fisher tomographic scale, and neurological impact and clinical prognosis were assessed using the Glasgow Coma Scale (GCS), World Federation of Neurological Surgeons (WFNS) severity scale, and the Hunt-Hess (HH) scale.

### Procedures

Serum vitamin D levels were measured using competitive electrochemiluminescence immunoassay (Liaison®, DiaSorin, Stillwater, Minnesota, USA), a test that correlates well with Liquid Chromatography coupled to Tandem Mass Spectrometry (LC–MS/MS), currently considered the gold standard.

### Vitamin D measurement

Vitamin D sufficiency was estimated by measuring 25-hydroxyvitamin D (25[OH]D or calcidiol) concentrations. The ideal serum concentration of 25(OH)D for bone health is controversial, with most experts agreeing that levels below 20 ng/mL are not ideal for bone health^55,56^. However, ideal serum concentrations of 25(OH)D for extra-skeletal health have not been well established yet. Given the controversy around ideal serum concentrations of vitamin D, the definitions of sufficiency, insufficiency, and deficiency of vitamin D are only approximate. In this study, we followed the Endocrine Society's 2011 guideline, where circulating concentrations of 25(OH)-D were divided into three subgroups to categorize vitamin D status in adults: deficient (< 20 ng/ml), insufficient (20–29 ng/ml), and sufficient (≥ 30 ng/ml), encompassing the concept of hypovitaminosis D, which includes both deficiency and insufficiency.

## Results

The clinical and demographic characteristics of the sample are shown in Table 1. A questionnaire was administered to 110 patients diagnosed with ruptured intracranial aneurysms. A total of 11 individuals were excluded, resulting in a sample of 99 patients. Among the excluded individuals, nine were excluded based on the exclusion criteria, and two were excluded due to centrifugation and storage failure of the biochemical vials for vitamin D analysis. The majority of individuals were female (79.8%) with a mean age of 53.22 ± 14.53 years. Most patients had a normal body mass index (BMI). Concerning comorbidities and risk factors, 10.1% of the sample had diabetes mellitus and 14.1% reported alcohol consumption. Systemic arterial hypertension was present in 54.5% of patients and 33.3% of the sample smoked.

**Table 1:** Demographic and clinical characteristics of the sample.

Indicators	Vit D < 30.0 ng/ml	Vit D ≥ 30.0 ng/ml	*p* value
Demographics			
Age (years)	53.85 ± 15.00 (53.00)	49.43 ± 10.91 (49.50)	*p*^a^ = 0.294
Female, n (%)	71 (71.7)	8 (8.08)	
Hypertension, n (%)	48 (48.5)	6 (6.06)	*p*^d^ = 0.343
Diabetes mellitus, n (%)	8 (8.08)	2 (2.02)	*p*^c^ = 0.630
Smoking, n (%)	28 (28.3)	5 (5.05)	*p*^c^ = 0.106
Alcohol use, n (%)	11 (11.1)	3 (3.03)	*p*^c^ = 0.413
BMI	25.58 ± 3.83 (25.22)	24.04 ± 3.51 (24.00)	*p*^a^ = 0.161
Solar exposure index	10.81 ± 8.68 (8.68)	11.97 ± 7.05 (13.37)	*p*^b^ = 0.418
Skin phototype			*p*^c^ = 0.552
I	3 (3.03)	–	
II	11 (11.1)	2 (2.02)	
III	22 (22.2)	1 (1.01)	
IV	32 (32.3)	7 (7.07)	
V	14 (14.1)	3 (3.03)	
VI	3 (3.03)	1 (1.01)	

^a^Using the Student’s *t* test with equal variances.

^b^Using the Mann–Whitney test.

^c^Using Fisher's exact test.

^d^Using Pearson's chi-square test.

*Statistically significant difference at the 5.0% level.

Regarding skin phototype, it is noteworthy that 60.6% of the sample had skin phototypes ranging from IV to VI, with skin phototype IV (39.4%) contributing the most, consistent with previous studies conducted on a similar population in Brazil^56^.

Although ideal serum values for vitamin D levels remain controversial, especially when considering different study populations, we chose a cutoff point for vitamin D sufficiency of serum values ≥ 30 ng/ml, as established by the Endocrine Society. Hypovitaminosis D was defined as serum values < 30 ng/ml. In our sample, a significant portion of individuals had vitamin D hypovitaminosis (85.9%), with a median of 19.9 ng/ml. This finding is impactful, especially when combined with the fact that most individuals had Fitzpatrick skin type IV or V and high rates of sun exposure (SI 9.03 P50). There was no difference in the solar index values between genders (males: 13.26 ± 10.26; females: 10.39 ± 7.9; *p* = 0.292).

The neurological characteristics of the sample are presented in Table 2. Regarding neurological prognosis scales for aSAH, the samples had a mean score of 2.37 ± 0.98 on the HH scale, 2.05 ± 1.27 on the WFNS scale, and 13.36 ± 2.50 on the GCS. The majority of the sample had a Fisher grade of IV (44.4%) on the cranial computed tomography, followed by Fisher grade III (35.35%).

**Table 2:** Neurological characteristics of the sample.

Indicators	Vit D < 30.0 ng/ml	Vit D ≥ 30.0 ng/ml	*p* value
Neurologics			
GCS	13.47 ± 2.46 (14.00)	12.71 ± 2.76 (13.50)	*p*^b^ = 0.338
Fisher	3.16 ± 1.03 (3.00)	2.64 ± 1.08 (3.00)	*p*^b^ = 0.054
HUNT HESS	2.38 ± 0.98 (2.00)	2.36 ± 1.01 (2.00)	*p*^b^ = 0.998
WFNS	2.02 ± 1.27 (1.00)	2.21 ± 1.31 (2.00)	*p*^b^ = 0.580
mRS			*p*^d^ = 0.384
< 3	44 (44.4)	9 (9.09)	
> 3	41 (41.4)	5 (5.05)	
Delayed cerebral ischemia			*p*^d^ = 0.840
Yes	34 (34.3)	6 (6.06)	
No	51 (51.5)	8 (8.08)	
Aneurysm location			*p*^c^ = 0.571
ICA	48 (48.5)	6 (6.06)	
MCA	22 (22.2)	4 (4.04)	
ACA	23 (23.2)	4 (4.04)	
PC	5 (5.05)	2 (2.02)	
Aneurysm size (mm)	6.65 ± 4.41	5.85 ± 2.11	*p*^b^ = 0.898

^a^Using the Student's *t* test with equal variances.

^b^Using the Mann–Whitney test.

^c^Using Fisher's exact test.

^d^Using Pearson's chi-square test.

^e^Using Kruskal Wallis test.

*Statistically significant difference at the 5.0% level.

When comparing the results of these scales and the serum level of vitamin D to assess patient prognosis, there was no significant correlation. In the prognostic evaluation, there was also no statistically significant correlation with hypovitaminosis D in relation to the presence of delayed cerebral ischemia (DCI).

Regarding the locations of ruptured aneurysms, the majority were in the internal carotid artery (ICA), followed by the anterior cerebral artery (ACA), middle cerebral artery (MCA), and posterior circulation (PC). There was no difference in the locations between individuals with hypovitaminosis and patients without vitamin D hypovitaminosis. Patients with hypovitaminosis D (< 30 ng/ml) had slightly larger aneurysms, but this finding was not statistically significant.

Therefore, although the sample comprises a significant portion of individuals with vitamin D hypovitaminosis, this status did not appear to impact the degree of clinical or neurological impairment resulting from aSAH.

As an additional finding, it was observed that the initial HH grade correlated with morbidity, being the main parameter, with the majority of patients with HH < 3 having a favorable outcome (ERm < 3; Table 3).

**Table 3:** Evaluation of the Hunt Hess score according to the mRS score.

HuntHess	mRS			
	< 3	> 3	Total	*p* value
	n (%)	n (%)	n (%)	
< 3	42 (42.9)	18 (18.4)	60 (61.2)	*p*^a^ < 0.001*
> 3	11 (11.2)	27 (27.6)	38 (38.8)	
Total	53 (54.1)	45 (45.9)	98 (100.0)	

^a^Using Pearson's chi-square test.

*Statistically significant difference at the 5.0% level.

Three patients in the sample died before the treatment of the ruptured aneurysm. With respect to treatment, 57.57% were treated with microsurgery, with 54 undergoing clipping and three undergoing bypass surgery, and 38 (38.4%) were treated with endovascular intervention (Table 4).

**Table 4:** Evaluation of Hunt Hess and mRS scores according to the aneurysm management.

Scores	Tratamento					*p* value
	Clipping	Coilling	Bypass	Pre-treatment mortality	Total	
	n (%)	n (%)	n (%)	n (%)	n (%)	
HuntHess						*p*^a^ = 0.859
< 3	33 (61.1)	24 (63.2)	1 (33.3)	2 (66.7)	60 (61.2)	
> 3	21 (38.9)	14 (36.8)	2 (66.7)	1 (33.3)	38 (38.8)	
Total	54 (100.0)	38 (100.0)	3 (100.0)	3 (100.0)	98 (100.0)	
mRS						*p*^a^ = 0.023*
< 3	33 (61.1)	17 (43.6)	3 (100.0)	–	53 (53.5)	
> 3	21 (38.9)	22 (56.4)	–	3 (100.0)	46 (46.5)	
Total	54 (100.0)	39 (100.0)	3 (100.0)	3 (100.0)	99 (100.0)	

^a^Using Fisher's exact test.

*Statistically significant difference at the 5.0% level.

## Discussion

Several studies in temperate climates have demonstrated both the seasonality of aSAH and vitamin D, which both show a higher prevalence during colder periods. During colder seasons, there is less sunlight exposure, which may result in reduced vitamin D activation^57^. To date, all studies of sSAH and vitamin D have been conducted in temperate climate countries. This study is the first to evaluate serum vitamin D levels in patients with ruptured cerebral aneurysms in a tropical region near the equator, specifically, northeastern Brazil.

### Demographic characteristics and endocrinological features

The sample consisted of 99 patients, which is a significant number and indicates a high demand at the hospital over an 12-month period. Most of the patients in the sample were female (79.8%) and middle-aged (53.22 ± 14.53 years old). These demographics are similar to those commonly described in populations with ruptured aneurysms^34–36^. Some well-established risk factors for aneurysm formation and rupture, such as smoking and hypertension, were present in a substantial portion of the sample (33.3% and 54.5%, respectively).

The study’s sample was from the state of Pernambuco, located in the northeastern region of Brazil, in a tropical climate zone at a low latitude (approximately 8° 3′ 15″ South). In this region, sunlight strikes the surface almost directly year-round, resulting in minimal variation in day and night duration. Unlike temperate climates, there are no well-defined seasons in this area. Therefore, the high levels of ultraviolet (UV) solar radiation (≥ 8) or extreme (≥ 11) are a characteristic feature throughout the year.

The Hospital da Restauração, where the data was collected, exclusively serves patients from the brazilian Unified Health System (SUS) and is a state reference center for neurology and neurosurgery, receiving a high annual volume of patients.

Most of the patients treated come from low-income backgrounds with limited education, many referred from rural areas where their primary occupation involves outdoor agricultural work, resulting in high sun exposure. Some studies have evaluated serum vitamin D levels in individuals from different professions, demonstrating that individuals who work indoors with limited sun exposure are at a higher risk of developing vitamin D deficiency. This is in contrast to the study's population, which should have lower risk due to significant sun exposure during their work^58–61^. Therefore, this study has a unique sample in terms of high solar index (SI) values.

Regarding ethnic characteristics, there was a high prevalence of skin phototypes with a high melanin concentration (Fitzpatrick skin type III or higher). This ethnic characteristic, common in northeastern Brazil, differs significantly from the populations in most previous studies, which were conducted in temperate climate regions^34–36^. Several studies have demonstrated that individuals with darker skin generally have lower absolute levels of vitamin D than Caucasians. Melanin's interference with vitamin D production activity can contribute to this phenomenon. Melanin competes for ultraviolet B (UVB) photons (220–390 nm) on the skin, hindering most of them from being used for the photolysis of 7-dehydrocholesterol (7-DHC) and the subsequent formation of pro-vitamin D3, the initial reaction of the classical pathway. Individuals with higher melanin concentration in their skin require longer UVB sun exposure to achieve the same serum level of 25-hydroxyvitamin D [25(OH)D] compared to those with lower melanin concentration. Some studies show that individuals from South Asia, who typically have skin phototype V, need long exposure periods (about 25–40 min) with a large area of exposed skin (35%) to reach average annual serum concentrations of 25 nmol/L, which is similar to natives of England who achieve such levels with significantly lower solar indexes^62^.

Moreover, it is important to note that the majority of vitamin D (about 80%) in the body is produced endogenously through sunlight exposure, as described earlier. Foods rich in vitamin D are primarily fatty fish from cold, deep waters or those containing ergosterol (vitamin D2) present in fungi. Neither form is easily accessible to the majority of the population, especially the one in this study, which is a predominantly low-income population. Furthermore, no patient in the study was using vitamin D supplementation.

Even in areas with high sun exposure, some studies have reported a high prevalence of vitamin D deficiency, indicating that sunlight exposure alone is insufficient to achieve adequate vitamin D levels in the majority of individuals^63^. Certain genetic variants may lead to different responses of serum 25-OHD to pro-vitamin D. Minor alleles of CYP2R1 (belonging to the cytochrome P450 subfamily IIR1 and encoding hepatic 25-hydroxylase), such as rs10500804, and specific group complement, encoding VDR and DBP, such as rs4588 and rs7041, are associated with a reduced response of serum 25-OHD to UV radiation. Consequently, there may be lower efficacy of sunlight exposure in preventing and treating vitamin D deficiency^64^.

In light of this, our primary hypothesis justifying the substantial prevalence of hypovitaminosis D even in areas with high sun exposure is the presence of polymorphisms in genes activating 25-OHD, likely constituting a natural selection characteristic favoring high sun exposure to avoid overproduction or intoxication.

Some studies have observed a high prevalence of vitamin D deficiency even in areas with high sun exposure and we particularly highlighting the research demonstrating hypovitaminosis D in a population from the same city with similar demographic characteristics as our current study^65^. The highlight of our paper is that it revealed even lower serum vitamin D levels in patients with SAH.

### Neurological characteristics

A high rate of hypovitaminosis D was observed in the sample of patients with ruptured aneurysms studied (85.9%). This finding aligns with recent studies that suggest that vitamin D deficiency could be associated with cerebral aneurysms^34–36^. A recent controlled study also demonstrated that vitamin D levels are independently associated with cerebral aneurysms^37^. Additionally, another study by the same authors demonstrated an association between hypovitaminosis D and aneurysm rupture^66^.

To characterize the neurological aspects of our sample of patients with aSAH, several classic scales were applied. The sample had an average score of 2.37 ± 0.98 on the HH scale, 2.05 ± 1.27 on the WFNS scale, and 13.36 ± 2.50 on the GCS. These scales play an important role in the prognosis of patients with aSAH. In our study, it was observed that the HH grade correlates with morbidity, with statistical significance when compared to the modified Rankin scale. This finding is consistent with other studies in the literature^67,68^.

Although there seems to be a relationship between hypovitaminosis D and aneurysm formation/rupture, there are few studies that demonstrate a relationship between the degree of vitamin D deficiency and patient prognosis. A recent study of 40 patients with aSAH showed a high prevalence of vitamin D deficiency among them, but no difference in clinical outcomes^36^.

In our study, when we compared the results of the prognostic scales for aSAH and the modified Rankin Scale with the levels of vitamin D deficiency (< 20 ng/ml), insufficiency (20 to < 30 ng/ml), or sufficiency (≥ 30 ng/ml), we also did not find a significant correlation with patient prognosis.

Regarding the correlation between the size of the aneurysm and vitamin D deficiency, some epidemiological studies reported a correlation between the size of extracranial artery dilation, aortic aneurysms, and vitamin D deficiency^69–74^. However, there are no studies yet demonstrating this correlation with cerebral aneurysms. In our study, we observed that patients with hypovitaminosis D (< 30 ng/ml) had slightly larger aneurysms compared to patients with sufficient vitamin D, but this finding did not reach statistical significance.

Regarding the locations of the ruptured aneurysms, the majority were in the internal carotid artery (ICA), followed by the anterior cerebral artery (ACA), middle cerebral artery (MCA), and posterior circulation (PC). There was no difference in the location between individuals with hypovitaminosis and those without hypovitaminosis D, although a detailed subgroup analysis was not conducted.

As for the presence of DCI, some studies provide evidence of an association between vitamin D deficiency, the frequency of cerebral vasospasms, and the overall outcome of patients after subarachnoid hemorrhage^36,75–77^. Additionally, the therapeutic utility of vitamin D has been demonstrated in animal models, leading to a reduction in the incidence of DCI, serving as a predictive indicator, a prevention method, and/or a treatment option after spontaneous SAH. However, these results have not yet been fully investigated in human studies^78,79^.

In our study, there was no statistically significant correlation between the presence of DCI and hypovitaminosis D. Similar results were obtained in a recent study of 33 patients, showing a higher proportion of patients with aSAH had vitamin D deficiency, but there was no difference in the occurrence of vasospasm or overall outcome^36^.

Furthermore, among the patients analyzed, three patients from the sample died before the treatment of the ruptured aneurysm. Regarding the treatment of the sample, 57.57% underwent microsurgery, with 54 undergoing clipping and 3 undergoing bypass technique, while 38 (38.4%) were treated with endovascular intervention (coilling). It was also observed that among the patients who underwent microsurgery, there was a better morbidity outcome assessed by the modified Rankin Scale. This could be attributed to the fact that the majority of these patients had a HH grade < 3, and the patients allocated for endovascular procedures were older.

### Study limitations

There are several limitations in our study. The COVID-19 pandemic was a limiting factor, primarily causing delays in data collection for over a year after approval by the Ethics Committee. Additionally, the pandemic and home isolation may have directly influenced the sunlight exposure of these patients in the months leading up to data collection.

The fact that this was an uncontrolled study conducted at a single center is another limitation. However, it's important to note that the study's original objective did not include control groups. On the other hand, this study's strength lies in its inclusion of a significant number of individuals (n = 99) from a low-latitude region, unlike most studies that were conducted in temperate climate regions.

To our knowledge, this is the first study to investigate the rate of vitamin D deficiency in patients with ruptured aneurysms in a tropical region, specifically in northeastern Brazil.

## Conclusion

Since this is only a descriptive study, a causal relationship cannot be established. Nevertheless, a significant percentage of a population exposed to high levels of sunlight and affected by aSAH had hypovitaminosis D.

Despite various studies showing a relationship between vitamin D deficiency, ruptured aneurysms, and delayed cerebral ischemia (DCI) in animals, there is still no scientific evidence justifying vitamin D supplementation in this patients.

Although recent studies demonstrate a high prevalence of vitamin D deficiency even in areas with high sun exposure, the highlight of our paper is that it revealed even lower serum vitamin D levels in patients with aSAH.

This is a broad topic, and there are still relatively few studies on it, especially in regions between the tropics. While there are limitations in the current studies, advances in research show a promising correlation between hypovitaminosis D and cerebral aneurysms. Therefore, more research should be conducted, both pre-clinical and clinical, to further investigate this relationship.

## Data Availability

The datasets used and/or analysed during the current study available from the corresponding author on reasonable request.

## References

[1] Crago EA, Price TJ, Bender CM, Ren D, Poloyac SM, Sherwood PR (2016). Impaired work productivity after aneurysmal subarachnoid hemorrhage. J. Neurosci. Nurs..

[2] Feigin VL, Rinkel GJE, Lawes CMM, Algra A, Bennett DA, van Gijn J (2005). Risk factors for subarachnoid hemorrhage: An updated systematic review of epidemiological studies. Stroke.

[3] Lawton MT, Vates GE (2017). Subarachnoid hemorrhage. N. Engl. J. Med..

[4] Osgood ML (2021). Aneurysmal subarachnoid hemorrhage: Review of the pathophysiology and management strategies. Curr. Neurol. Neurosci. Rep..

[5] Silva JME (2007). Brief history of rickets and of the discovery of vitamin D. Acta Reumatol. Port..

[6] Slominski AT, Kim T-K, Shehabi HZ, Semak I, Tang EKY, Nguyen MN, Benson HAE, Korik E, Janjetovic Z, Chen J, Yates CR, Postlethwaite A, Li W, Tuckey RC (2012). In vivo evidence for a novel pathway of vitamin D3 metabolism initiated by P450scc and modified by CYP27B1. FASEB J..

[7] Slominski AT, Li W, Kim TK, Semak I, Wang J, Zjawiony JK, Tuckey RC (2015). Novel activities of CYP11A1 and their potential physiological significance. J. Steroid Biochem. Mol. Biol..

[8] Slominski A, Kim TK, Li W (2015). Detection of novel CYP11A1-derived secosteroids in the human epidermis and serum and pig adrenal gland. Sci. Rep..

[9] Slominski RM, Raman C, Elmets C, Jetten AM, Slominski AT, Tuckey RC (2021). The significance of CYP11A1 expression in skin physiology and pathology. Mol. Cell Endocrinol..

[10] Slominski, A. T., Tuckey, R. C., Jetten, A. M. & Holick, M. F. Recent advances in vitamin D biology: Something new under the sun. *J. Investig. Dermatol.* S0022-202X(23)02426-0 (2023). 10.1016/j.jid.2023.07.003 PMC10841303 37791933

[11] Gouni-Berthold I, Berthold HK (2021). Vitamin D and vascular disease. Curr. Vasc. Pharmacol..

[12] Eyles DW, Smith S, Kinobe R, Hewison M, McGrath JJ (2005). Distribution of the vitamin D receptor and 1 alpha-hydroxylase in human brain. J. Chem. Neuroanat..

[13] Cui X, Pelekanos M, Liu P-Y, Burne THJ, McGrath JJ, Eyles DW (2013). The vitamin D receptor in dopamine neurons; its presence in human substantia nigra and its ontogenesis in rat midbrain. Neuroscience.

[14] Merrigan SL, Kennedy BN (2017). Vitamin D receptor agonists regulate ocular developmental angiogenesis and modulate expression of dre-miR-21 and VEGF: Vitamin D regulates ocular angiogenesis, miR-21 and VEGF. Br. J. Pharmacol..

[15] Norman PE, Powell JT (2005). Vitamin D, shedding light on the development of disease in peripheral arteries. Arterioscler. Thromb. Vasc. Biol...

[16] Rosen CJ, Adams JS, Bikle DD, Black DM, Demay MB, Manson JE (2012). The nonskeletal effects of vitamin D: An Endocrine Society scientific statement. Endocr. Rev..

[17] Bouillon, R. Extra-skeletal effects of vitamin D. *Em: Front. Hormone Res.* 72–88 (2018). 10.1159/000486072 29597236

[18] Van De Luijtgaarden KM, Mt V, Se H, Chon-Chol M (2012). Vitamin D deficiency may be an inde- pendent risk factor for arterial disease. Eur. J. Vasc. Endovasc. Surg..

[19] Rai V, Agrawal DK (2017). Role of vitamin D in cardiovascular diseases. Endocrinol. Metab. Clin. N. Am..

[20] Lin R, Amizuka N, Sasaki T, Aarts MM, Goltzman H (2002). 25-Dihydroxyvitamin D3 promotes vascularization of the chondro-osseous junction by stimulating expression of vascular endothelial growth factor and matrix metalloproteinase 9. J. Bone Miner. Res..

[21] Martorell S, Hueso L, Gonzalez-Navarro H, Collado A, Sanz M-J, Piqueras L (2016). Vitamin D receptor activation reduces angiotensin-II–induced dissecting Abdominal aortic aneurysm in apolipoprotein E–knockout mice. Arterioscler. Thromb. Vasc. Biol..

[22] Walters MR, Wicker DC, Riggle PC (1986). 1,25-Dihydroxyvitamin D3 receptors identified in the rat heart. J. Mol. Cell Cardiol..

[23] O’Connell TD, Simpson RU (1996). Immunochemical identification of the 1,25-dihydroxyvitamin D3 receptor protein in human heart. Cell Biol. Int..

[24] Chen S, Law CS, Grigsby CL, Olsen K, Hong T-T, Zhang Y (2011). Cardiomyocyte-specific deletion of the vitamin D receptor gene Results in cardiac hypertrophy. Circulation.

[25] Carthy W, Ooi BS (1989). 25-Dihydroxyvita- min D3 and rat vascular smooth muscle cell growth. Hypertension.

[26] Somjen D, Weisman Y, Kohen F, Gayer B, Limor R, Sharon O (2005). 25-Hydroxyvitamin D 3–1α-hydroxylase is expressed in human vascular smooth muscle cells and is upregulated by parathyroid hormone and estrogenic compounds. Circulation.

[27] Merke J, Milde P, Lewicka S, Hügel U, Klaus G, Mangelsdorf DJ (1989). Identification and regulation of 1,25-dihydroxyvitamin D3 receptor activity and biosynthesis of 1,25-dihydroxyvitamin D3. Studies in cultured bovine aortic endothelial cells and human dermal capillaries. J. Clin. Investig..

[28] Guillot X, Semerano L, Saidenberg-Kermanac’h N, Falgarone G (2010). Boissier M-C Vitamin D and inflammation. Joint Bone Spine.

[29] Silvagno F, De Vivo E, Attanasio A, Gallo V, Mazzucco G, Pescarmona G (2010). Mitochondrial localization of vitamin D receptor in human platelets and differentiated megakaryocytes. PLoS ONE.

[30] Ginde AA, Scragg R, Schwartz RS, Camargo CA (2009). Prospective study of serum 25-hydroxyvitamin D level, cardiovascular disease mortality, and all-cause mortality in older U.S. adults: Vitamin D and mortality. J. Am. Geriatr. Soc..

[31] Giallauria F, Tanaka TMY, Maggio M, Canepa M, Elango P (2012). Arterial stiffness and vitamin D levels: The Baltimore longitudinal study of aging. J. Clin. Endocrinol. Metab..

[32] Pilz S (2012). Vitamin D and cardiovascular disease: Update and outlook. Scand. J. Clin. Lab. Investig..

[33] Manson JE, Buring JE, Vitamin D, VITAL Research Group (2019). Supplements and prevention of cancer and cardiovascular disease. N. Engl. J. Med..

[34] Guan J, Karsy M, Eli I, Bisson EF, McNally S, Taussky P (2016). Increased incidence of hypovitaminosis D among patients requiring treatment for cerebral aneurysms. World Neurosurg..

[35] Randhawa TS, Dhandapani S, Singla N, Maskara P, Sachdeva N, Gupta SK (2019). Vitamin D deficiency in aneurysmal subarachnoid hemorrhage. J. Cerebro Vasc. Sci..

[36] Alvarado Y, Perez A, Rodriguez-Vega G (2015). Effects of vitamin D deficiency in aneurysmal subarachnoid hemorrhage. Crit. Care Med..

[37] Wei S, Yuan X, Fan F, Guo X-B, Guan S (2021). The relationship between the level of vitamin D and ruptured intracranial aneurysms. Sci. Rep..

[38] Lips P, Duong T, Oleksik A, Black D, Cummings S, Cox D (2001). A global study of vitamin D status and parathyroid function in postmenopausal women with osteoporosis: Baseline data from the multiple outcomes of raloxifene evaluation clinical trial. J. Clin. Endocrinol. Metab..

[39] Klingberg E, Oleröd G, Konar J, Petzold M, Hammarsten O (2015). Seasonal variations in serum 25-hydroxy vitamin D levels in a Swedish cohort. Endocrine.

[40] Backes D, Rinkel GJE, Algra A, Vaartjes I, Donker GA, Vergouwen MDI (2016). Increased incidence of subarachnoid hemorrhage during cold temperatures and influenza epidemics. J. Neurosurg..

[41] Schröder-Heurich B, von Hardenberg S, Brodowski L, Kipke B, Meyer N, Borns K (2019). Vitamin D improves endothelial barrier integrity and counteracts inflammatory effects on endothelial progenitor cells. FASEB J..

[42] Ashouri R, Fangman M, Brielmaier J, Fields ZA, Campo N, Doré S (2021). Nutritional supplementation of naturally occurring vitamin D to improve hemorrhagic stroke outcomes. Front. Neurol..

[43] PubMed. US National Library of Medicine. National Institutes of Health: https://pubmed.ncbi.nlm.nih.gov/?term=vitamin+D+%5Btitle%5D. Search on: Aug.05 2023.

[44] Christakos S, Dhawan P, Verstuyf A, Verlinden L, Carmeliet G (2016). Vitamin D: Metabolism, molecular mechanism of action, and pleiotropic effects. Physiol. Rev..

[45] Bouillon R, Marcocci C, Carmeliet G, Bikle D, White JH, Dawson-Hughes B (2019). Skeletal and extraskeletal actions of vitamin D: Current evidence and outstanding questions. Endocr. Rev..

[46] Cui X, Gooch H, Groves NJ, Sah P, Burne TH, Eyles DW (2015). Vitamin D and the brain: Key questions for future research. J. Steroid. Biochem. Mol. Biol..

[47] Izzo M, Carrizzo A, Izzo C, Cappello E, Cecere D, Ciccarelli M (2021). Vitamin D: Not just bone metabolism but a key player in cardiovascular diseases. Life (Basel).

[48] Szejko N, Acosta JN, Both CP, Leasure A, Matouk C, Sansing L (2022). Genetically-proxied levels of vitamin D and risk of intracerebral hemorrhage. J. Am. Heart Assoc..

[49] Wang W, Li Y, Meng X (2023). Vitamin D and neurodegenerative diseases. Heliyon.

[50] Pál É, Ungvári Z, Benyó Z, Várbíró S (2023). Role of vitamin D deficiency in the pathogenesis of cardiovascular and cerebrovascular diseases. Nutrients.

[51] Wei S, Yuan X, Li D, Fan F, Guo X, Xu Y (2022). Vitamin D level is associated with rupture of intracranial aneurysm in patients with subarachnoid hemorrhage. Front. Neurol..

[52] Roberts WE (2009). Skin type classification systems old and new. Dermatol. Clin..

[53] Sachdeva S (2009). Fitzpatrick skin typing: Applications in dermatology. Indian J. Dermatol. Venereol. Leprol..

[54] Barger-Lux MJ, Heaney RP (2002). Effects of above average summer sun exposure on serum 25-hydroxyvitamin D and calcium absorption. J. Clin. Endocrinol. Metab..

[55] Giustina A, Adler RA, Binkley N, Bouillon R, Ebeling PR, Lazaretti-Castro M (2019). Controversies in vitamin D: Summary statement from an international conference. J. Clin. Endocrinol. Metab..

[56] Giustina A, Bouillon R, Dawson-Hughes B, Ebeling PR, Lazaretti-Castro M, Lips P (2022). Vitamin D in the older population: A consensus statement. Endocrine.

[57] Mishra S, Mamourian A (2017). Letter to the Editor. Seasonal subarachnoid hemorrhage: Temperature or daylight?. J. Neurosurg..

[58] Sowah D, Fan X, Dennett L, Hagtvedt R, Straube S (2017). Vitamin D levels and deficiency with different occupations: A systematic review. BMC Public Health.

[59] Brouwer-Brolsma EM, Vaes AMM, van der Zwaluw NL, van Wijngaarden JP, Swart KMA, Ham AC (2016). Relative importance of summer sun exposure, vitamin D intake, and genes to vitamin D status in Dutch older adults: The B-PROOF study. J. Steroid Biochem. Mol. Biol..

[60] Nakamura K, Nashimoto M, Hori Y, Muto K, Yamamoto M (1999). Serum 25-hydroxyvitamin D levels in active women of middle and advanced age in a rural community in Japan. Nutrition.

[61] Bachhel R, Singh NR, Sidhu JS (2015). Prevalence of vitamin D deficiency in north-west Punjab population: A cross-sectional study. Int. J. Appl. Basic Med. Res..

[62] Webb AR, Kazantzidis A, Kift RC, Farrar MD, Wilkinson J, Rhodes LE (2018). Colour counts: Sunlight and skin type as drivers of vitamin D deficiency at UK latitudes. Nutrients.

[63] Bahlous A, Krir A, Mrad M, Bouksila M, Kalai S, Kilani O, El KEC, Sahli H, Laadhari N (2022). Vitamin D in healthy Tunisian population: Preliminary results. J. Med. Biochem..

[64] Petersen RA, Larsen LH, Damsgaard CT (2017). Common genetic variants are associated with lower serum 25-hydroxyvitamin D concentrations across the year among children at northern latitudes. Br. J. Nutr..

[65] Azevedo M, Bandeira L, Luza C, Lemos A, Bandeira F (2018). Vitamin D deficiency, skin phototype, sun index, and metabolic risk among patients with high rates of sun exposure living in the tropics. J. Clin. Aesthet. Dermatol..

[66] Sharma A, Sharma JK (2020). Association of bone mineral density, Vitamin D, and serum calcium in intracranial aneurysm. Asian J. Neurosurg..

[67] Medani K, Sadan O, Martin KS, Binongo JN, Samuels OB (2017). Association between Hunt and Hess grade and the modified Rankin scale among patients with non-traumatic subarachnoid hemorrhage. Neurocrit. Care.

[68] Lahiri S, Kamel H, Meyers EE, Falo MC, Al-Mufti F, Schmidt JM (2016). Patient-powered reporting of modified Rankin Scale outcomes via the internet. Neurohospitalist.

[69] Mayer O, Filipovský J, Seidlerová J, Vaněk J, Dolejšová M, Vrzalová J (2012). The association between low 25-hydroxyvitamin D and increased aortic stiffness. J. Hum. Hypertens..

[70] Takagi H, Umemoto T (2017). Vitamins and abdominal aortic aneurysm. Int. Angiol..

[71] Krishna SM (2019). Vitamin D as A protector of arterial health: Potential role in peripheral arterial disease formation. Int. J. Mol. Sci..

[72] Demir M, Uyan U, Melek M (2012). The relationship between vitamin D deficiency and thoracic aortic dilatation. Vasa.

[73] Wong YYE (2013). Is hypovitaminosis D associated with abdominal aortic aneurysm, and is there a dose-response relationship?. Eur. J. Vasc. Endovasc. Surg. Off. J. Eur. Soc. Vasc. Surg..

[74] Wong MSK, Leisegang MS, Kruse C, Vogel J, Schürmann C, Dehne N (2014). Vitamin D promotes vascular regeneration. Circulation.

[75] Fiani B, Barthelmass M, Siddiqi I, Kortz M, Pennington E, Pasko K (2022). Vitamin D as a modifiable risk factor, predictor, and theoretical therapeutic agent for vasospasm in spontaneous subarachnoid hemorrhage. Acta Neurol. Belg..

[76] Kashefiolasl S, Leisegang MS, Helfinger V, Schürmann C, Pflüger-Müller B, Randriamboavonjy V (2021). Vitamin D-A new perspective in treatment of cerebral vasospasm. Neurosurgery.

[77] Poole KES, Loveridge N, Barker PJ, Halsall DJ, Rose C, Reeve J (2006). Reduced vitamin D in acute stroke. Stroke.

[78] Enkhjargal B, McBride DW, Manaenko A, Reis C, Sakai Y, Tang J (2017). Intranasal administration of vitamin D attenuates blood-brain barrier disruption through endogenous upregulation of osteopontin and activation of CD44/P-gp glycosylation signaling after subarachnoid hemorrhage in rats. J. Cereb. Blood Flow Metab..

[79] Enkhjargal B, Malaguit J, Ho WM, Jiang W, Wan W, Wang G (2019). Vitamin D attenuates cerebral artery remodeling through VDR/AMPK/eNOS dimer phosphorylation pathway after subarachnoid hemorrhage in rats. J. Cereb. Blood Flow Metab..

